# Antimicrobial resistance needs to be combated at primary levels of prevention by nurses

**DOI:** 10.1002/nop2.445

**Published:** 2020-02-09

**Authors:** Sarah Oerther, Daniel B. Oerther

**Affiliations:** ^1^ School of Nursing Saint Louis University Saint Louis MO USA; ^2^ King’s College London London UK

Antimicrobial resistance (AMR) is a threat to public health (O'Neil, 2014; World Health Organization, 2018). AMR occurs when antimicrobial therapies become ineffective at killing infections caused by bacteria, fungi, parasites and viruses, and this leads to drug‐resistant infections (World Health Organization, 2018). Drug‐resistant infections are impacting all populations (Centers for Disease Control, 2018; Logan & Bonomo, 2016; World Health Organization, 2018; Zerr et al., 2014). Inappropriate use of antimicrobial therapies is believed to be a significant contributing factor to the emergence and spread of AMR (O'Neil, 2014; World Health Organization, 2018).

Drug‐resistance infections contribute to longer hospital stays, higher medical costs and increased incidence of morbidity (World Health Organization, 2018; Zetts, Stoesz, Smith, & Hyun, 2018). Though there is advocacy for the prevention of AMR, interventions have enjoyed limited success because of primary care providers’ fears and patient's perceived demand for pills to treat all illnesses (Martínez‐González et al., 2017; Wood et al., 2012). For instance, primary care providers want to meet the patients’ need to feel satisfied with their care (Martínez‐González et al., 2017; Wood et al., 2012; Zetts et al., 2018). Primary care providers also may fear that patients may become ill without the use of antimicrobial therapies (Martínez‐González et al., 2017; Wood et al., 2012; Zetts et al., 2018). AMR needs to be combated with primary levels of prevention by nurses.

## 1 BACKGROUND

In the United States (US), 23,000 deaths and two million illnesses were caused by antibiotic‐resistant bacterial infections (Centers for Disease Control, 2018). If AMR continues to rise, it is estimated that by 2050 up to 10 million people will die annually from drug‐resistant infections (O'Neil, 2014). The indirect economic losses attributed to AMR could result in a global loss of 60–100 trillion USD between now and 2050 (O'Neil, 2014).

One of the main risk factors for AMR in the United States is inappropriate prescription of antimicrobial therapies by primary care providers **(**Zetts et al., 2018)**.** Researchers have found that approximately 154 million primary care visits in the United States result in an antibiotic prescription and approximately 30 per cent, or 47 million, of these prescriptions are not necessary (PEW, 2016).

Researchers have shown that patients’ demand for antimicrobial therapies varies depending on cultural background and geographic location (Larson et al., 2009; Morgan & Hart, 2009; Zetts et al., 2018). Researchers have found Hispanic cultures request antibiotics more frequently (Larson et al., 2009). This could be because antibiotics are available over‐the‐counter in some Latin American countries (Larson et al., 2009). Geographically, southern states in the United States average 920 prescriptions per 1,000 people compared to western states in the United States where residents receive an average of 632 antibiotic prescriptions per 1,000 people **(**Zetts et al., 2018)**.** However, people in extremely rural communities in the United States tend to wait longer before seeing a provider for treatment for upper respiratory symptoms (Morgan & Hart, 2009). Researchers have found that people in rural communities are more accepting of a provider's recommendations to treat an infection with supportive care rather than a prescription for antibiotics (Morgan & Hart, 2009).

## 2 PRIMARY PREVENTION

Interventions at the primary level are needed long term to prevent the spread of AMR (Centers for Disease Control, 2018; Logan & Bonomo, 2016; World Health Organization, 2018; Zerr et al., 2014). Nursing interventions at the primary level need to focus on overcoming existing barriers to care, including gaps in knowledge about best practices, patients’ expectations for antibiotics, the need to see patients in a short amount of time and primary care providers’ concerns regarding patient satisfaction when they do not prescribe an antimicrobial therapy (Sanchez, Fleming‐Dutra, Roberts, & Hicks, 2016).

Appropriate nursing interventions at the primary level are also needed to overcome cultural and geographic barriers (Hicks et al., 2015). For example, people living in rural communities are less likely to listen to AMR prevention campaigns (Hicks et al., 2015). Researchers believe this barrier exists because prevention campaigns do not incorporate rural cultural considerations (Hicks et al., 2015). Patients living in rural areas need educational programmes designed to address rural social norms (Hicks et al., 2015).

## 3 CONCLUSION

Researchers have demonstrated that the primary level of prevention of AMR has a clear impact and is cost‐effective (Ball, Dains, Flynn, Solomon, & Stewart, 2019; Hersh, Jackson, & Hicks, 2013). Implementing primary prevention needs further development by nurses to decrease the number of people receiving unnecessary antimicrobial therapies from primary care providers (Ball et al., 2019; Hersh et al., 2013). Nurses also need to create appropriate health education programmes about AMR to improve patents’ knowledge and practices towards safe use antimicrobial therapies, especially in rural areas and among Latino populations in the United States (Larson et al., 2009; Zetts et al., 2018).

## AUTHOR CONTRIBUTIONS

All authors contributed equally to this article.
